# Supplementary material for: Resonance-enhanced spectral funneling in Fabry–Perot resonators with a temporal boundary mirror
Source: Nanophotonics. 2022 Jan 12;11(9):2045–55. doi: 10.1515/nanoph-2021-0667 (PMC11501943; doi:10.1515/nanoph-2021-0667)
Supplement: Supplementary file 1 — Supplementary Material [file j_nanoph-2021-0667_suppl.docx]

**Supplementary material: Resonance-enhanced spectral funneling in Fabry–Perot resonators with a temporal boundary mirror**

1. Characterization of the temporal mirror

The temporal and spectral transmission properties of the temporal mirror (i.e., an optically pumped GaAs substrate) are shown in Fig. S1. To illustrate the step-like time-varying behavior of the temporal mirror upon ultrafast excitation, we plot the peak field of the transmitted THz pulse through the temporal mirror as a function of the pump delay in Fig. S1a (here, the positive pump delay corresponds to the situation after the temporal boundary). As seen in the plot, the transmission reduced by the pump pulse excitation was maintained up to a pump delay of 100 ps with a very slow recovery rate. Figure S1b shows the amplitude transmission spectra through the temporal mirror after the temporal boundary (measured at three different pump delays, 4 ps, 50 ps and 100 ps). The measured amplitude transmission spectra show slight dispersiveness in the frequency range of interest [1], which justifies the assumption made regarding the temporal mirror in the TCMT calculations.


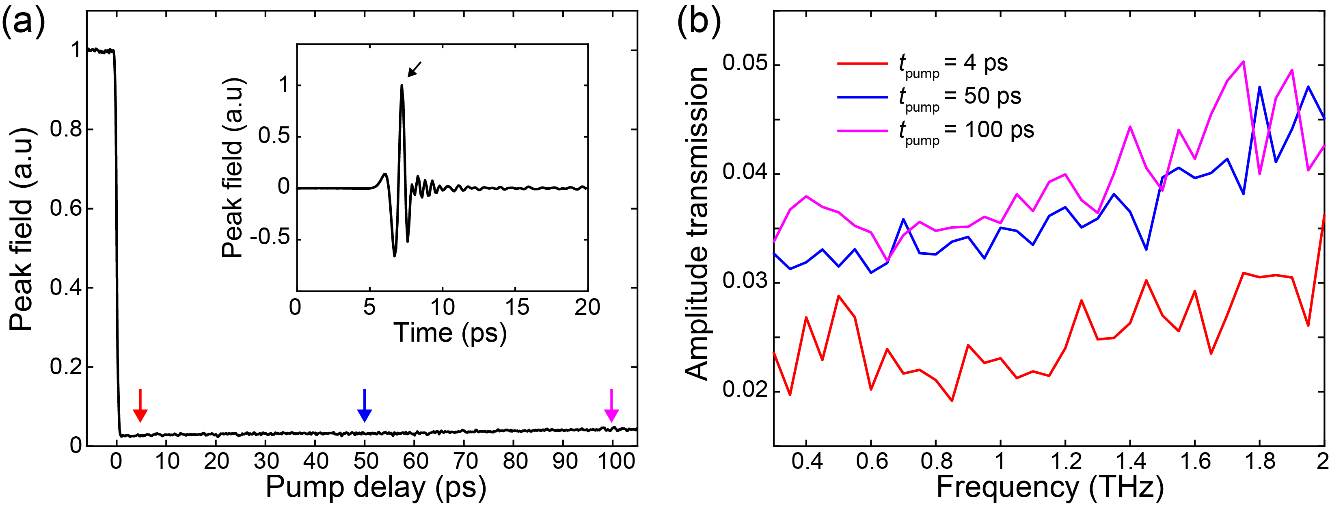


Fig. S1. Optical-pump/THz-probe characterization of the GaAs substrate. (a) The peak field of the transmitted THz pulse through the GaAs substrate is plotted as a function of the time delay between the near-infrared pump and THz probe pulses. The inset shows the representative waveform of a THz probe pulse with its peak field position indicated by an arrow. (b) Amplitude transmission spectra through the temporal mirror measured at *t*_pump_ = 4 ps, *t*_pump_ = 50 ps, and *t*_pump_ = 100 ps.

1. Characterization of the static mirror

The spectral characterization of the static mirror (i.e., a thin polyimide film patterned with an array of gold wires) is shown in Fig. S2. The measured transmission through the static mirror, $t\left( \omega\right)$, is plotted with crosses in Fig. S2a. From the measured spectra $t\left( \omega\right)$, the effective surface conductivity of the static mirror, $\sigma\left( \omega\right)$, can be estimated as follows [2]:

$$\sigma\left( \omega\right)=\frac{1}{Z_{0}}\left[ \frac{2}{t\left( \omega\right)}-2 \right],$$

where $Z_{0}$ is the impedance of free space. In Fig. S2b, the estimated complex-valued conductivity of the static mirror is plotted as a function of frequency. Additionally, the fitted surface conductivity (red and blue lines) by the Drude model, from which the amplitude transmission $t\left( \omega\right)$ can be fitted (a black line in Fig. S2a) are also shown. In the TCMT calculations presented in the main manuscript, the fitted amplitude transmission spectra of the static mirror were used to reproduce the measured resonance-enhanced spectral funneling behavior.


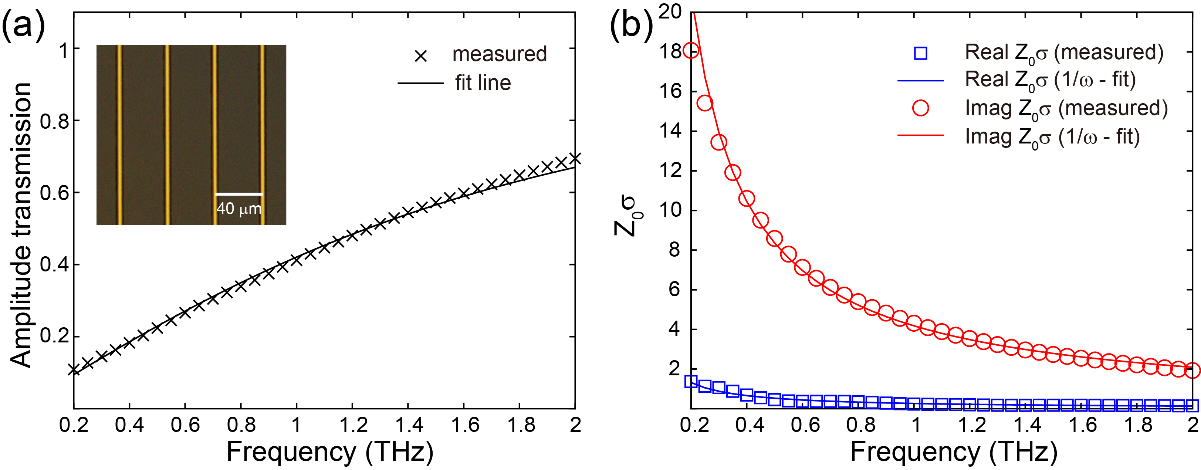


Fig. S2. (a) Amplitude transmission through the static mirror plotted as a function of frequency. The inset shows a microscopic image of the fabricated static mirror. The measured amplitude transmission spectrum is drawn with black crosses, and the Drude model fitting is drawn with a black line. (b) Complex-valued surface conductivity of the static mirror plotted as a function of frequency. Extracted data are drawn with scatters, while the fitted data are drawn with lines.

1. Reflection properties of static Fabry–Perot resonators

As the photoconductive layer formed on the surface of the GaAs substrate and the static mirror are much thinner than the wavelength of the input THz pulse, we can apply the following boundary condition to estimate the amplitude reflection spectra from the measured amplitude transmission spectra of the temporal and static mirrors [2]:

${\tilde{\boldsymbol{E}}}_{\boldsymbol{i}}\boldsymbol{(\omega)+}{\tilde{\boldsymbol{E}}}_{\boldsymbol{r}}\boldsymbol{(\omega)-}{\tilde{\boldsymbol{E}}}_{\boldsymbol{t}}\boldsymbol{(\omega)=0}$,

where ${\tilde{\boldsymbol{E}}}_{\boldsymbol{i}}\boldsymbol{(\omega)}$, ${\tilde{\boldsymbol{E}}}_{\boldsymbol{r}}\boldsymbol{(\omega)}$, and ${\tilde{\boldsymbol{E}}}_{\boldsymbol{t}}\boldsymbol{(\omega)}$ are the spectral amplitudes of the incident, reflected and transmitted THz pulses, respectively. The estimated amplitude reflection spectra, ${\tilde{\boldsymbol{E}}}_{\boldsymbol{r}}\boldsymbol{(\omega)}\boldsymbol{/}{\tilde{\boldsymbol{E}}}_{\boldsymbol{i}}\boldsymbol{(\omega)}$, for the temporal and static mirrors are plotted in Fig. S3.


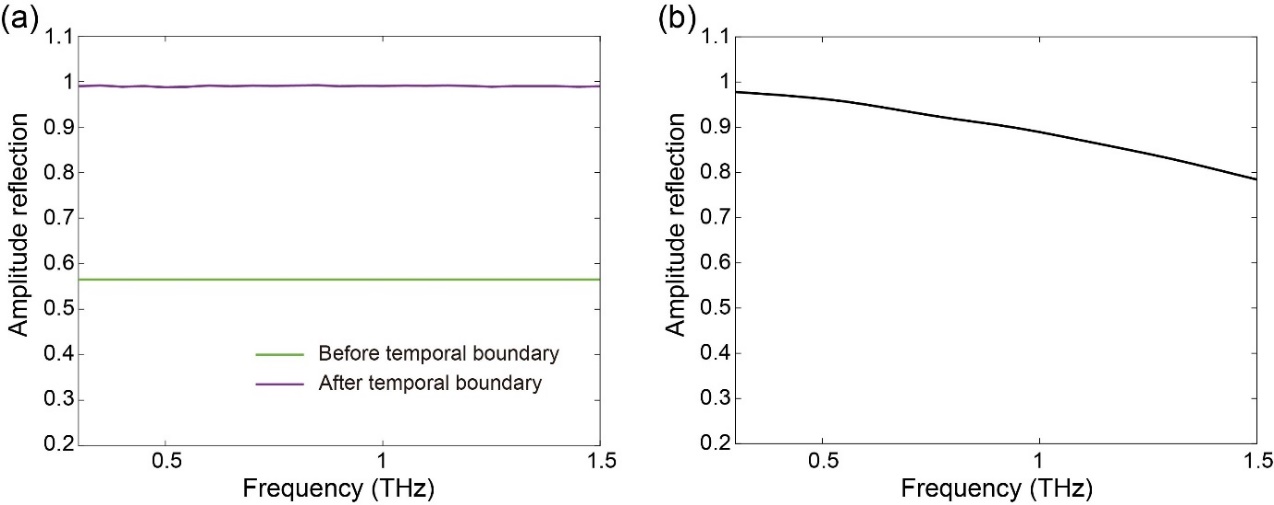


Fig. S3. (a) Estimated amplitude reflection spectra of the temporal mirror. The amplitude reflection spectrum (green line) is obtained by assuming a situation prior to the temporal boundary, while the spectrum (purple line) is obtained by assuming a situation after the temporal boundary. (b) Estimated amplitude reflection spectrum of the static mirror.

With the amplitude reflection spectra of constituting mirrors, those of FP resonators with cavity lengths of 250 μm (Fig. S4a-S4c) and 900 μm (Fig. S4d-S4e) can be estimated by considering multiple interferences [3]. In these calculations, the GaAs substrate was assumed to be semi-infinite (i.e., a half-space filled with GaAs). Here, for simplicity, the incident and reflected THz pulses were assumed to propagate in the GaAs substrate. As seen in Fig. S4a and d, the amplitude reflection spectra of the FP resonators prior to the temporal boundary are characterized by low-Q Fabry–Perot resonances, especially due to the low amplitude reflection of the temporal mirror (see Fig. S3a). On the other hand, after the temporal boundary, the incident THz pulse can barely be coupled to the FP resonator and is almost totally reflected (see Fig. S4a and d). The waveforms of the reflected THz pulses can also be calculated by performing an inverse Fourier transform of the product of the amplitude reflection spectrum and the incident THz pulse spectrum. Fig. S4b and d show that the waveforms reflected from the FP resonators prior to the temporal boundary are characterized by the interference of multiple pulse trains. In contrast, the waveforms reflected from the FP resonators posterior to the temporal boundary are similar to those almost totally reflected as predicted (Fig. S4c and d).


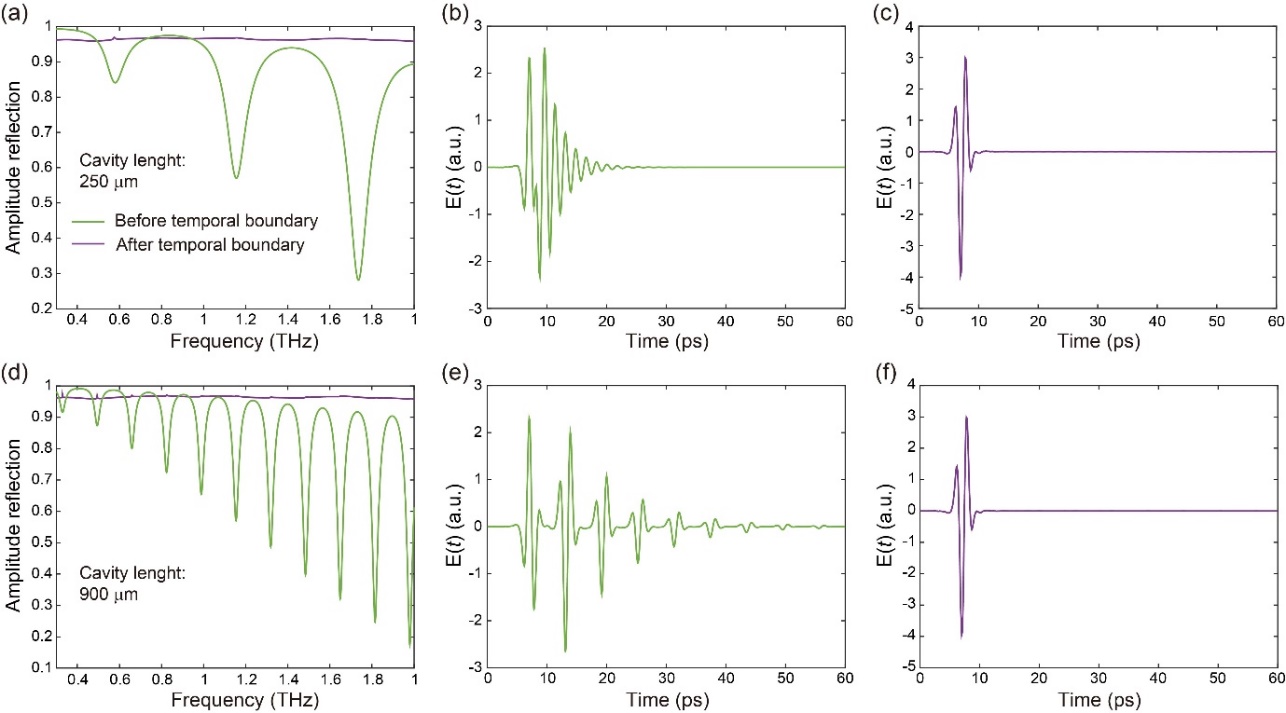


Fig. S4. (a-c) Calculated reflection properties of the Fabry–Perot resonator with a cavity length of 250 μm. (a) Amplitude reflection spectra from the static Fabry–Perot resonators. The green line denotes the amplitude reflection spectrum prior to the temporal boundary, while the purple line denotes the spectrum posterior to the temporal boundary. (b) Calculated waveform reflected from the static Fabry–Perot resonator prior to the temporal boundary. (c) Calculated waveform reflected from the static Fabry–Perot resonator after the temporal boundary. (d-f) Calculated reflection properties of the Fabry–Perot resonator with a cavity length of 900 μm.

References

[1] D. Grischkowsky, S. Keiding, M. van Exter, and Ch. Fattinger, “Far-infrared time-domain spectroscopy with terahertz beams of dielectrics and semiconductors,” *J. Opt. Soc. Am. B*, vol.7, pp.2006–2015, 1990.

[2] M. C. Nuss, and J. Orenstein, “Millimeter and Submillimeter Wave Spectroscopy of Solids,” *Top. Curr. Chem*, vol.74, pp.7-50, 1998.

[3] E. Hecht, “Optics,” 4th ed., Chapter. 9.6, Addison Wesley, 2002.
